# The Ndc80 complex targets Bod1 to human mitotic kinetochores

**DOI:** 10.1098/rsob.170099

**Published:** 2017-11-15

**Authors:** Katharina Schleicher, Michael Porter, Sara ten Have, Ramasubramanian Sundaramoorthy, Iain M. Porter, Jason R. Swedlow

**Affiliations:** Centre for Gene Regulation and Expression, School of Life Sciences, University of Dundee, Dundee, UK

**Keywords:** chromosome segregation, kinetochore, BOD1, PP2A inhibitor, Ndc80 complex, Knl1

## Abstract

Regulation of protein phosphatase activity by endogenous protein inhibitors is an important mechanism to control protein phosphorylation in cells. We recently identified Biorientation defective 1 (Bod1) as a small protein inhibitor of protein phosphatase 2A containing the B56 regulatory subunit (PP2A-B56). This phosphatase controls the amount of phosphorylation of several kinetochore proteins and thus the establishment of load-bearing chromosome-spindle attachments in time for accurate separation of sister chromatids in mitosis. Like PP2A-B56, Bod1 directly localizes to mitotic kinetochores and is required for correct segregation of mitotic chromosomes. In this report, we have probed the spatio-temporal regulation of Bod1 during mitotic progression. Kinetochore localization of Bod1 increases from nuclear envelope breakdown until metaphase. Phosphorylation of Bod1 at threonine 95 (T95), which increases Bod1's binding to and inhibition of PP2A-B56, peaks in prometaphase when PP2A-B56 localization to kinetochores is highest. We demonstrate here that kinetochore targeting of Bod1 depends on the outer kinetochore protein Ndc80 and not PP2A-B56. Crucially, Bod1 depletion functionally affects Ndc80 phosphorylation at the N-terminal serine 55 (S55), as well as a number of other phosphorylation sites within the outer kinetochore, including Knl1 at serine 24 and 60 (S24, S60), and threonine T943 and T1155 (T943, T1155). Therefore, Ndc80 recruits a phosphatase inhibitor to kinetochores which directly feeds forward to regulate Ndc80, and Knl1 phosphorylation, including sites that mediate the attachment of microtubules to kinetochores.

## 1. Introduction

To preserve genome integrity, the two sister chromatids of each mitotic chromosome must be distributed equally between daughter cells. Movement of sister chromatids to opposite poles of a dividing cell requires attachment to spindle microtubules of opposing orientation. Errors in the attachment process can lead to chromosome missegregation and aneuploidy (i.e. an aberrant number of chromosomes). Aneuploid karyotypes are the major cause of spontaneous miscarriages in humans [1] and often observed in cancer genomes [2]. A multi-complex protein interface between mitotic chromosomes and the spindle apparatus called the kinetochore is responsible for both the establishment and regulation of the microtubule attachment process [3]. The kinetochore consists of approximately 30 core structural proteins that are arranged into several functional subcomplexes. Structural kinetochore proteins constitute the physical link between chromosomes and spindle microtubules. They also act as a signalling platform by recruiting checkpoint proteins, kinases and phosphatases. When a cell enters mitosis, kinase activity destabilizes kinetochore–microtubule interactions [4,5] to allow for dynamic kinetochore–microtubule interactions and thus prevention of attachment errors [6]. Conversely, phosphatase activity is needed at later stages of the attachment process to stabilize kinetochore–microtubule interactions that have passed quality control. At anaphase onset, dephosphorylation of kinetochore proteins helps maintain load-bearing attachments [7]. There is a conserved, dynamic system comprised at least eight kinases and two phosphatases that control microtubule–kinetochore attachments [3,8].

The detailed role and function of protein phosphatases and their interplay at the kinetochore is only beginning to be elucidated [9–11], but both are of great interest as they are absolutely required to ensure faithful chromosome segregation. Protein phosphatase 1 (PP1) dephosphorylates microtubule-binding kinetochore proteins to ultimately stabilize attachments [12,13]. However, recruitment of PP1 to kinetochores requires the initial activity of protein phosphatase 2A (PP2A) [14,15], highlighting the importance of coordinated timely activation of these kinetochore components. PP2A is a heterotrimeric enzyme, composed of a scaffolding (A) subunit, catalytic (C) subunit and a regulatory (B) subunit [16]. It is targeted to the kinetochore by the B56 family of B subunits [11,17,18]. The highest mitotic occupancy of PP2A-B56 at kinetochores is reached in prometaphase and can be maximized by increasing the number of unattached kinetochores with nocodazole [11]. Under the same conditions, PP1 localization to kinetochores is low [12], suggesting that PP2A accumulation at unattached kinetochores alone is not sufficient to recruit PP1 and that additional molecular signals are required to activate PP2A-mediated PP1 recruitment in metaphase.

We have recently identified a small kinetochore protein, Biorientation defective 1 (Bod1), that can specifically inhibit PP2A-B56 [9,19]. Bod1 is required for cognitive function in humans and *Drosophila* models [20]. Depletion of Bod1 from HeLa cells leads to premature loss of phosphorylation on several kinetochore proteins, including MCAK and CENP-U/PBIP1, due to unregulated activity of PP2A-B56, which causes an increase in aberrant chromosome attachments and defective chromosome segregation. Bod1 has also recently been shown to alleviate premature, radiation-induced chromatid separation in human lung and renal cell carcinoma cells, protecting against genomic instability [21]. Bod1, together with CIP2A [22], FAM122A [23], I1PP2A/ANP32A [24], I2PP2A/SET [25], TIP [26] and Arpp-19/Ensa [27,28], forms part of a growing family of PP2A inhibitors that have important roles in supporting cell division. However, little is known about the temporal localization of these PP2A regulators or how they modulate the activity of PP2A towards different substrates.

Here, we have studied the temporal recruitment and phospho-regulation of Bod1 at mitotic kinetochores. We show that Bod1 kinetochore targeting depends on the outer kinetochore protein Ndc80 (Nuclear division cycle protein 80, also known as highly expressed in cancer protein Hec1). Furthermore, we show that Bod1 can protect phosphorylation of a key site in the N-terminal tail of Ndc80 that is required for microtubule attachment, as well as several sites in Knl1, another outer kinetochore protein. These data further refine our understanding of how PP2A activity at the kinetochore is regulated and identify additional targets of the Bod1 phosphatase inhibitor pathway.

## 2. Results

### 2.1. Bod1 localizes to kinetochores throughout mitosis and is maximally phosphorylated in prometaphase

**Figure 1.**
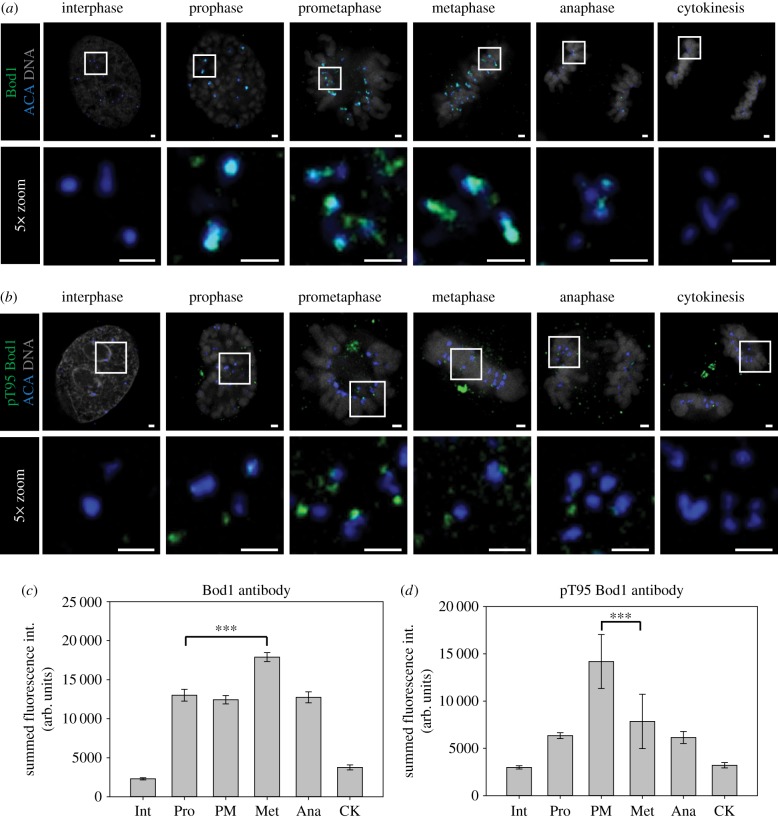
Cell cycle profiles of Bod1 kinetochore recruitment and phosphorylation. HeLa cells were fixed in paraformaldehyde and stained with (*a*) a pan-specific Bod1 peptide antibody or (*b*) a phospho-specific pT95 Bod1 peptide antibody (both green). Cells were co-stained with markers for the centromeric region (ACA, blue) and DNA (DAPI, grey). Top panel shows a single *z*-section of each cell cycle stage. The lower panels are magnifications of the same cell (section indicated by white boxes). Scale bars are 1 µm. Quantification of (*c*) total Bod1 or (*d*) phospho-T95 Bod1 fluorescence intensity at the kinetochore corresponding to experiments shown in (*a*) and (*b*). Three asterisks indicate high significance (*p* < 0.001) in multiple comparison after ANOVA on ranks. *n* = 10 cells per mitotic phase. Error bars represent standard error. Int, interphase; Pro, prophase; PM, prometaphase; Met, metaphase; Ana, anaphase; CK, cytokinesis.

To dissect the temporal regulation of Bod1 recruitment to kinetochores, we raised peptide antibodies for immunofluorescence profiling in HeLa cells. This antibody stains the kinetochore and staining is largely ablated by Bod1 siRNA treatment (figure 1*a*; electronic supplementary material, figure S1). We were especially interested in Bod1's role at the kinetochore, and so we quantified Bod1 kinetochore intensities within a 4-pixel (0.32 µm) radius of anti-centromere antibody (ACA) staining (figure 1*c*). Bod1 is first detected on kinetochores at nuclear envelope breakdown and reaches maximum occupancy at metaphase.

We showed previously that inhibition of PP2A-B56 by Bod1 is greatly enhanced when Bod1 is phosphorylated at T95 [9]. We therefore raised a phospho-specific antibody against this site (figure 1*b*; electronic supplementary material, figure S1). Quantification of pT95 Bod1 at kinetochores revealed that this post-translational modification peaks in prometaphase, before maximal recruitment of the total protein (figure 1*d*). This phosphorylation is reversed by Cdk1 inhibition (electronic supplementary material, figure S1*g,h*). PP2A-B56 levels at kinetochores are highest in prometaphase when attachments are weak [11]. The phosphorylation of Bod1 at T95 therefore coincides with the recruitment of PP2A-B56, consistent with a role in inhibiting PP2A-B56 activity and enabling correction of attachment errors in early mitosis.

### 2.2. Bod1 recruitment to kinetochores is independent of PP2A-B56 and Knl1

**Figure 2.**
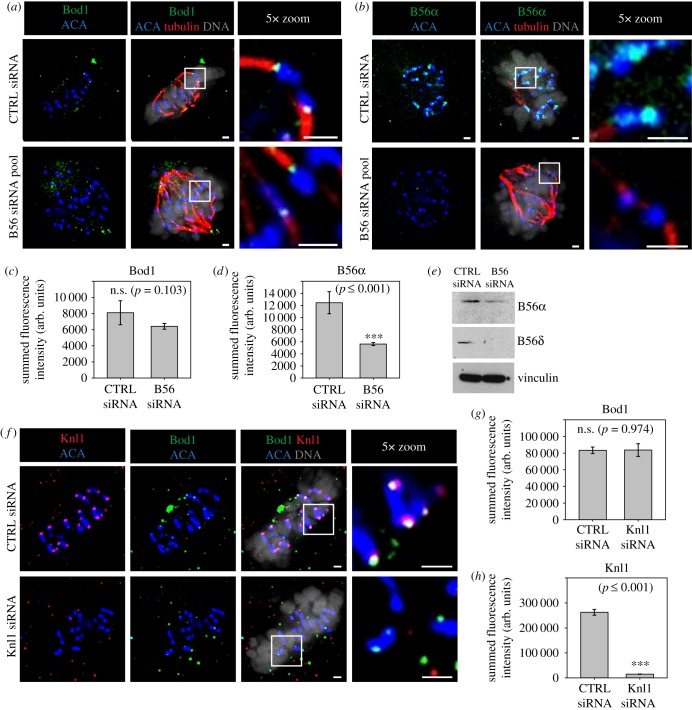
PP2A-B56 and Knl1 are dispensable for Bod1 recruitment to kinetochores. HeLa cells were treated with either control siRNA or a smart pool siRNA targeting all PP2A-B56 isoforms as described previously [11]. After 48 h cells were fixed in paraformaldehyde and stained with (*a*) the total Bod1 peptide antibody or (*b*) a PP2A-B56α isoform-specific antibody. Metaphase control cells or B56siRNA-treated cells showing the characteristic B56-depletion phenotype of metaphase chromosome alignment defects were imaged. The rightmost panels are magnifications of the same cell (region indicated by white boxes). Total (*c*) Bod1 and (*d*) B56α intensities at kinetochores were quantified. (*e*) Immunoblot corresponding to the B56-depletion experiments in (*a–d*) using antibodies against two of the five targeted B56 isoforms and vinculin as a loading control. (*f*) HeLa cells were treated with either control or Knl1 siRNA for 48 h, fixed in paraformaldehyde, and co-stained with both total Bod1 peptide antibody and a Knl1-specific antibody. Metaphase cells were imaged for both treatment conditions. The rightmost panels are magnifications of the same cell (section indicated by white boxes). Total (*g*) Bod1 and (*h*) Knl1 intensities at kinetochores were quantified. Single *z*-sections are shown for all images. Scale bars are 1 µm. Pairwise comparisons were evaluated by unpaired Student's *t*-test. Two-tailed *p*-values are shown. *n* = 10 cells per condition. Error bars represent standard error.

PP2A-B56 is a well-characterized component of the kinetochore with binding sites at both the outer kinetochore [17,18,29] and the inner centromere [30–32] (electronic supplementary material, figure S2). To test whether Bod1 and PP2A-B56 are co-recruited to kinetochores, we depleted PP2A-B56 from HeLa cells using a pool of B56 isoform-specific siRNAs [11] and quantified total Bod1 protein at the kinetochores (figure 2*a–e*). Surprisingly, there was no significant change in Bod1 recruitment to kinetochores upon B56 depletion.

Since it is difficult to achieve complete knockdown of B56 isoforms via siRNA (figure 2*e*), we then depleted the outer kinetochore protein Knl1, a structural kinetochore protein implicated in PP2A-B56 kinetochore targeting. Knl1 provides a binding platform for mitotic checkpoint proteins such as BubR1 [33,34]. BubR1 can bind the B56 subunit and thus mediate recruitment of a pool of PP2A-B56 to the outer kinetochore [17,18,29]. As with B56 depletion, siRNA-mediated knockdown of Knl1 did not affect Bod1 recruitment to kinetochores (figure 2*f–h*). We therefore conclude that Bod1 is recruited to kinetochores independently of PP2A-B56 and via a different interaction platform.

### 2.3. The mitotic interactome of Bod1 contains many outer kinetochore proteins including Ndc80

**Figure 3.**
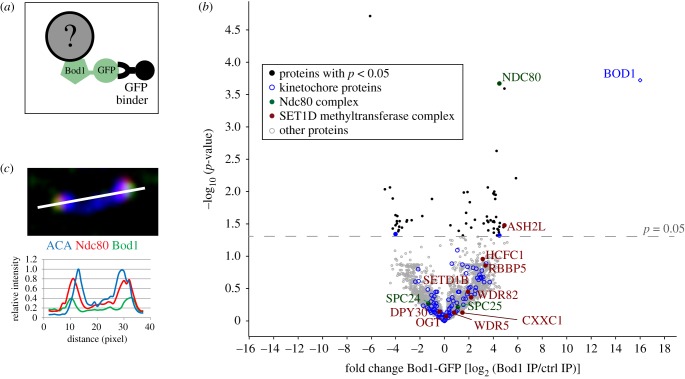
Proteomics analysis of mitotic Bod1-GFP affinity purifications. (*a*) GFP binder was used to affinity purify Bod1-GFP and associated proteins from mitotic HeLa cell lysates as described previously [9]. (*b*) Volcano plot of the mitotic Bod1 interactome detected by label-free quantification shotgun mass spectrometry. For each quantified protein, the –log_10_ of the *p*-value obtained in an unpaired Student's *t*-test with a threshold *p*-value of 0.05 is plotted against the fold change in Bod1-GFP samples versus control (represented by log_2_ of their intensity ratios). All kinetochore proteins detected are highlighted in blue. Components of the Ndc80 complex are highlighted in green and labelled. Components of the Set1b complex are highlighted in maroon and labelled. *n* = 4 biological replicates. (*c*) HeLa cells were fixed in paraformaldehyde and stained for Ndc80 (red), Bod1 (green) and a marker of the centromeric region (ACA, blue). A pair of kinetochores is shown. White line indicates the region chosen for a line profile (bottom panel) of fluorescence intensities across the kinetochore pair.

To discover candidate proteins that might target Bod1 to kinetochores we combined affinity purification of Bod1 with label-free quantitative mass spectrometry (MS) (figure 3; electronic supplementary material, figure S3). In mitotic lysates from HeLa cells expressing Bod1-GFP, we identified and quantified 3512 proteins. Of these, 42 were significantly enriched in affinity purifications from Bod1-GFP expressing cells compared to cells expressing GFP alone as a control (*n* = 4 biological replicates; electronic supplementary material, table S1). Gene ontology (GO) term analysis identified 95 centromere- and kinetochore-associated proteins in the Bod1-GFP affinity purifications (electronic supplementary material, table S2). Of these, Bod1 itself, Ndc80 and dynein intermediate chain 1 were significantly enriched in Bod1-GFP affinity purifications compared to controls (figure 3*b*; electronic supplementary material, figure S4*a*). The most reproducible kinetochore interactor was Ndc80; it was found in all four biological replicates of the experiment. Furthermore, of all kinetochore proteins detected, Ndc80 exhibited the highest fold change in Bod1-GFP affinity purifications compared to controls. Intensity analysis of the centromeric region in HeLa cells, co-stained with Bod1 and Ndc80 antibodies, revealed that immunofluorescence signals of the two proteins overlap at the outer kinetochore (figure 3*c*). The mitotic Bod1 interactome also contained components of the SET1B methyltransferase complex, with significant enrichment of ASH2 L. This is consistent with previous interaction results obtained in asynchronous HeLa cells [35].

### 2.4. Bod1 associates with the Ndc80 complex

**Figure 4.**
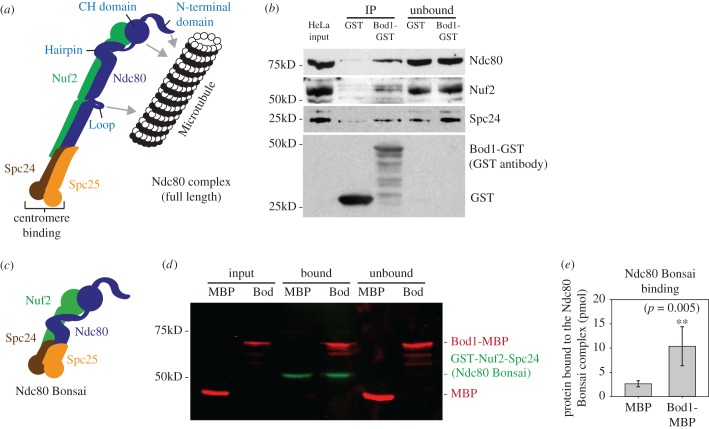
The Ndc80 complex interacts with Bod1 in mitotic HeLa cell extracts and *in vitro*. (*a*) Graphic representation of the full-length Ndc80 complex. Domains of interest, including the microtubule and centromere binding regions of the complex, are labelled. (*b*) Co-precipitation of Ndc80 complex components Ndc80, Nuf2 and Spc24 from mitotic HeLa cell extracts with purified Bod1-GST. (*c*) Representation of the recombinant Ndc80 Bonsai complex (as described in [38]). Ndc80–Spc25 and GST–Nuf2–Spc24 are expressed as fusion proteins. Both fusion constructs are co-expressed in *E. coli* from a dual-expression vector. (*d*) Of note, 150 pmol recombinant Ndc80 Bonsai, consisting of dimers of one Ndc80–Spc25 fusion protein with one GST–Nuf2–Spc24 fusion protein, was immobilized on Sepharose beads and incubated with 1 nmol Bod1-MBP or MBP. Binding was allowed for 1 h. Proteins were resolved by SDS-PAGE and immunoblotted using simultaneous detection of the MBP (red) epitope tag on Bod1 and the GST (green) epitope tag on Nuf2–Spc24. (*e*) Amount of bound protein in (*d*) was quantified relative to the input. Two asterisks indicate level of significance (*p* < 0.01) in unpaired Mann–Whitney rank sum test. *n* = 9 separate experiments. Error bars represent standard error.

To confirm the Bod1–Ndc80 interaction detected by MS analysis, we performed pull down assays with purified Bod1-GST on Sepharose beads to validate Ndc80 as a bona fide Bod1 interactor. Ndc80 localizes to kinetochores as part of the heterotetrameric Ndc80 complex, consisting of Ndc80, Nuf2, Spc24 and Spc25 [36,37] (figure 4*a*). Bod1-GST coated beads pulled out Ndc80, Nuf2 and Spc24 from mitotic HeLa cell lysates (figure 4*b*) (Spc25 was not tested). In order to determine whether this was a direct interaction with the complex, we tethered recombinant Ndc80 Bonsai, a truncated form of the Ndc80 complex containing a GST–Nuf2–Spc24 fusion and an Ndc80–Spc25 fusion that can be co-expressed in bacteria [38], to beads and incubated them with recombinantly expressed Bod1-MBP or MBP alone (figure 4*c,d*). Bod1-MBP interacted strongly with purified recombinant Ndc80 Bonsai complexes (figure 4*e*), supporting the proteomics and immunofluorescence data. Within the Ndc80 complex, Bod1-MBP preferentially bound to the Ndc80/Nuf2–GST dimer over the Spc24–GST/Spc25 dimer (electronic supplementary material, figure S5*b,c*). Together these results demonstrate Bod1 is part of the outer kinetochore and associates with the Ndc80 complex.

### 2.5. Ndc80 is essential for Bod1 kinetochore recruitment

**Figure 5.**
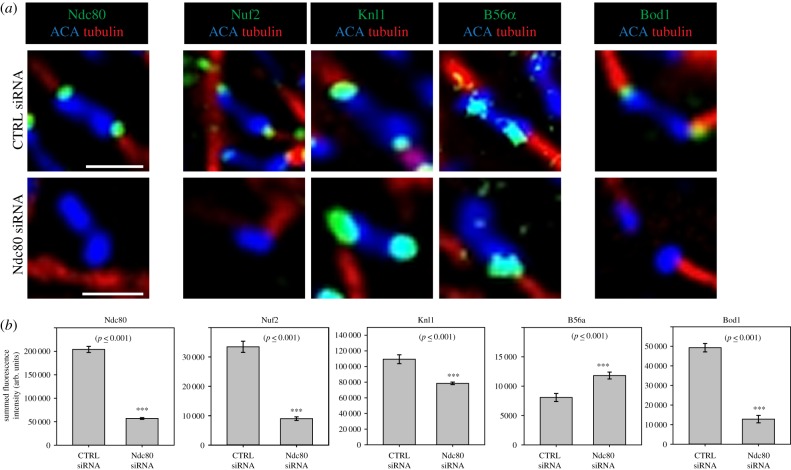
The Ndc80 complex is essential for Bod1 recruitment to mitotic kinetochores. (*a*) HeLa cells were treated with either control siRNA or Ndc80 siRNA. After 48 h cells were fixed in paraformaldehyde and stained with the indicated kinetochore proteins (green). Kinetochores of chromosomes on the metaphase plate within a single *z*-section are shown. Localization to the kinetochore—i.e. the interface between centromeric region (ACA, blue) and spindle microtubules (tubulin, red)—was compared. Scale bars are 1 µm. (*b*) Quantification of the images in (*a*). Pairwise comparisons were evaluated by unpaired Student's *t*-test. Two-tailed *p*-values are shown. *n* = 10 cells per condition. Error bars represent standard error.

To test if the Ndc80 complex was necessary for Bod1 kinetochore recruitment in cells, we depleted Ndc80 from HeLa cells using siRNA. Ndc80 depletion also reduced the immunofluorescence signal of its direct binding partner Nuf2 (figure 5). By contrast, we observed only a minor reduction in Knl1 signal, indicating that Ndc80 siRNA did not destabilize the entire outer kinetochore. Crucially, Ndc80 depletion resulted in significant loss of Bod1 from kinetochores, concomitant with an increase in localization of B56. The increase in B56 localization recapitulates our previous observations that siRNA depletion of Bod1 elevates B56 levels at kinetochores [9] and suggests that the localization of Bod1 to kinetochores might limit PP2A-B56 accumulation at these sites.

### 2.6. Bod1 depletion affects both PP2A-B56 recruitment and Knl1 phosphorylation

**Figure 6.**
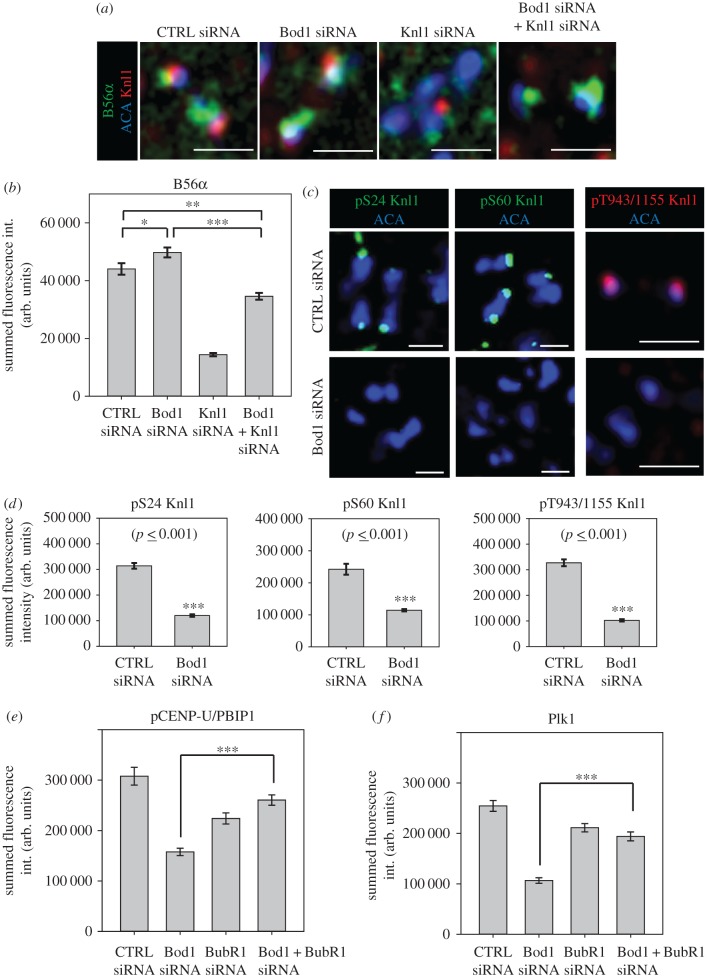
Bod1 controls Knl1 phosphorylation and antagonizes BubR1-dependent phosphatase activity at the kinetochore. (*a*) HeLa cells were treated with the indicated siRNAs for 48 h, fixed in paraformaldehyde and stained with a PP2A-B56α isoform-specific antibody (green) as well as a Knl1 antibody (red), and ACA (blue) as a centromeric marker. (*b*) Quantification of B56α kinetochore staining in the experiment shown in (*a*). Asterisks indicate degree of significance in multiple comparison after ANOVA on ranks: *p-value < 0.05, **p-value < 0.01, ***p-value < 0.001. (*c*) HeLa cells were treated with the indicated siRNAs for 48 h, fixed in paraformaldehyde and stained with different phospho-specific antibodies raised against three types of phosphorylatable motifs in Knl1: the SILK motif (S24), the RVSF motif (S60), and the MELT motifs (T943 and T1155). (*d*) Quantification of indicated Knl1 phosphosites in control and Bod1-depleted cells. Pairwise comparisons were evaluated by unpaired Student's *t*-test. Two-tailed *p*-values are shown. *n* = 10 cells per condition. Error bars represent standard error. Quantification of (*e*) phospho-CENP-U/PBIP1 and (*f*) Plk1 kinase staining at mitotic kinetochores of cells treated with the indicated siRNAs for 48 h. Three asterisks indicate high significance (*p* < 0.001) in multiple comparison after ANOVA on ranks. *n* = 10 cells per mitotic phase. Error bars represent standard error. All images shown are single *z*-sections. Scale bars are 1 µm.

A pool of PP2A-B56 is recruited to kinetochores through Knl1-bound checkpoint proteins [17,18,29,33,34]. Accordingly, Knl1 depletion led to a marked decrease in PP2A-B56α levels (figure 6*a,b*). We have previously shown that siRNA depletion of Bod1 leads to an increase in PP2A-B56α at kinetochores [9]. Therefore, we wanted to determine if this Bod1-dependent increase of PP2A-B56 levels at kinetochores could be prevented by co-depletion of Knl1. Surprisingly, co-depletion of Bod1 and Knl1 resulted in PP2A-B56α levels that were intermediate between those observed in kinetochores depleted of either Knl1 or Bod1 alone. This suggests that Bod1-regulated PP2A recruitment partially depends on Knl1, although there might also be a Knl1-independent mechanism to recruit PP2A-B56 to kinetochores.

To investigate whether the Knl1-associated PP2A-B56 pool was regulated by Bod1, we examined serine 24 and serine 60 phosphorylation sites within the N-terminus of Knl1, which are regulated by Aurora B and PP2A-B56 [12,15]. Upon Bod1 depletion, we observed a significant reduction of Knl1 phosphorylation at both S24 within the SILK motif and S60 within the RVSF motif (figure 6*c,d*), suggesting that increased PP2A-B56 activity in Bod1-depleted kinetochores directly affects Knl1 phosphorylation.

Dephosphorylation of SILK and RVSF domains increases PP1 binding to Knl1, which in turn leads to dephosphorylation of the MELT motifs within Knll [15,39]. Using an antibody that recognizes phospho-T943 and phospho-T1155 within two of the Knl1 MELT motifs we examined the levels of phospho-MELT staining in Bod1-depleted cells. We observed a 60% reduction in phosphorylation of these epitopes in Bod1-depleted cells (figure 6*c,d*). While complete loss of MELT phosphorylation leads to ablation of the spindle assembly checkpoint [39–41], the intact checkpoint response and checkpoint protein recruitment observed in Bod1-depleted cells [19] suggests that the remaining phospho-MELT epitopes are sufficient to maintain a checkpoint response in HeLa cells.

We previously demonstrated that Bod1 also controls phosphorylation on CENP-U/PBIP1 and other kinetochore proteins distal from Knl1 [9,19]. To determine if these phosphorylation sites were dependent on Knl1-bound PP2A-B56, we co-depleted Bod1 and BubR1, and measured phospho-CENP-U/PBIP1 staining. Bod1 and BubR1 co-depletion significantly rescued CENP-U/PBIP1 phosphorylation when compared to Bod1 depletion alone (figure 6*e*). We also observed a small drop in CENP-U/PBIP phosphorylation upon BubR1 depletion alone. This is probably due to loss of BubR1-bound Plk1 [42,43] and changes to mitotic progression upon loss of BubR1 [44]. Phosphorylated CENP-U/PBIP1 provides a kinetochore docking site for Plk1 [45]. Therefore, Plk1 docking is dramatically reduced in Bod1 siRNA-depleted cells. However, we observed significantly increased Plk1 kinetochore levels in Bod1 and BubR1 co-depleted cells (figure 6*f*). Taken together, these results suggest that Bod1 regulates the activity of PP2A-B56 bound to the Knl1/checkpoint protein complex, and this not only affects Knl1 phosphorylation directly, but also the association of Plk1 with the kinetochore.

### 2.7. Bod1 depletion results in loss of Ndc80 phosphorylation at its N-terminal tail

**Figure 7.**
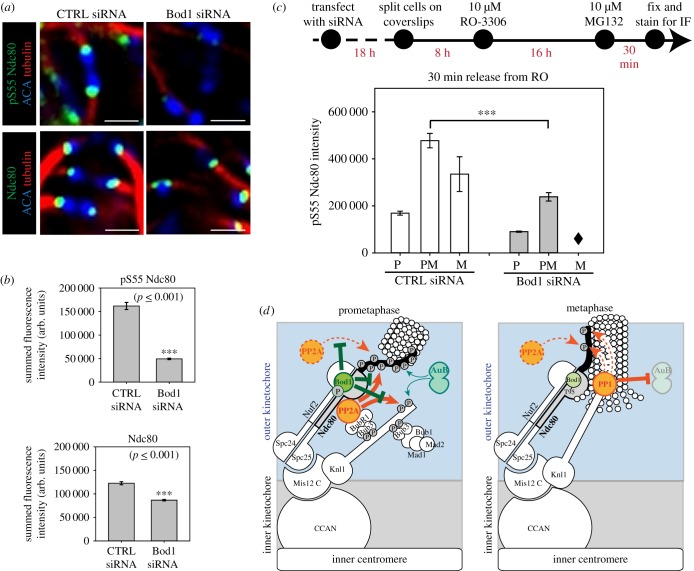
Bod1 depletion interferes with phosphorylation of the Ndc80 N-terminal tail. (*a*) HeLa cells were treated with control or Bod1 siRNA for 48 h, fixed in paraformaldehyde and stained with a phospho-specific antibody against a phosphoepitope (pS55) in the Ndc80 N-terminal tail, or a total Ndc80 antibody. Metaphase cells or cells with the characteristic Bod1 chromosome misalignment phenotype were imaged. Kinetochores of chromosomes on the metaphase plate within a single z-plane are shown. Scale bars are 1 µm. (*b*) pS55 and total Ndc80 intensities at kinetochores were quantified. Pairwise comparisons were evaluated by unpaired Student's *t*-test. Two-tailed *p*-values are shown. *n* = 10 cells per condition. Error bars represent standard error. (*c*) Timeline and quantification of treatments to compare kinetics of Ndc80 phosphorylation in control and Bod1-depleted HeLa cells in early mitosis. Twenty-six hours after transfection, cells were synchronized at G2/M transition with the Cdk1 inhibitor RO-3306 for 16 h. They were then released into culture medium containing the proteasome inhibitor MG132 to prevent mitotic exit and fixed 30 min after their release from G2/M arrest. pS55 Ndc80 fluorescence intensities at kinetochores of mitotic cells were quantified. *n* = 50 mitotic cells per condition. Three asterisks indicate high significance in pairwise multiple comparison after ANOVA on ranks (*p* < 0.001). Diamond indicates that no quantification could be performed for metaphase pS55 Ndc80 in Bod1 siRNA-treated cells, because lack of Bod1 caused a significant delay in early mitotic progression leading to the absence of metaphase cells 30 min after release from RO-3306. (*d*) A model integrating data presented in this paper with previous findings on the temporal regulation of kinetochore phosphatases in mitosis. Bod1 phosphorylation in early mitosis can inhibit PP2A-B56 activity [9] and prevent premature dephosphorylation of Ndc80 at its N-terminus and Knl1 at the SILK, RVSF and MELT motifs. Dephosphorylation of Bod1 in later stages by an unknown mechanism, coincides with increased PP2A activity and recruitment of PP1. PP2A, bound at an unidentified site within the kinetochore, and PP1 are free to dephosphorylate kinetochore epitopes and inhibit Aurora B activity in metaphase [12–14,48–50]. Dashed lines indicate implied activity but no direct evidence.

Bod1 depletion by siRNA leads to mitotic arrest as cells are unable to maintain chromosome alignment in metaphase until anaphase onset [19]. This biorientation phenotype is accompanied by an increase in syntelic kinetochore–microtubule attachments, an attachment conformation in which a pair of sister kinetochores connects to spindle microtubules emanating from the same pole. Such a form of attachment can lead to erroneous mitosis, aneuploidy and cell death [46], and therefore needs to be corrected before cells progress through mitosis. Correction of syntelic attachments is enabled by phosphorylation of outer kinetochore proteins [4,5,7,47], among them the N-terminus of Ndc80. Upon phosphorylation, the affinity of these proteins to microtubules is reduced and attachments are destabilized [5]. As our data suggest that Bod1 both associates with the Ndc80 complex and regulates the phosphorylation of different phosphoepitopes, we wanted to evaluate if Ndc80 phosphorylation is also dependent on Bod1 levels. We probed Ndc80 phosphorylation at its N-terminal serine 55 (S55) in Bod1-depleted cells using a phospho-specific antibody against this site. First, we compared control metaphase cells with Bod1-depleted cells that exhibited a clear biorientation phenotype. Bod1-depleted cells with biorientation phenotypes exhibited a 65% decrease in Ndc80 phosphorylation at S55 (figure 7*a,b*). However, Bod1 depletion and development of the biorientation phenotype also led to a small but significant reduction (approx. 30%) in the intensity of total Ndc80 protein at the kinetochore. We therefore aimed to understand the effects of Bod1 depletion on Ndc80 phosphorylation at S55 in cells just entering mitosis, before development of the mature chromosome misalignment phenotype.

To selectively sample cells between nuclear envelope breakdown and metaphase, we first synchronized cells at the G2/M transition with RO-3306, a Cdk1 inhibitor [51]. The cells were then released into mitosis. Their medium was supplemented with the proteasome inhibitor MG132 to limit mitotic progression to prophase, prometaphase, and metaphase (figure 7*c*). Thirty minutes after RO-3306 release we assessed both Ndc80 pS55 kinetochore intensities and mitotic progression. At the early stages of mitosis, when error correction takes place, Bod1-depleted cells had significantly lower Ndc80 pS55 levels than control cells (figure 7*c*). Bod1-depleted cells also showed a delay in mitotic progression, even in these early stages of mitosis. After 30 min of release from the RO-3306 mediated G2/M block, the population of Bod1siRNA-treated cells had only progressed to prophase or prometaphase. None of the cells had entered metaphase, which is consistent with previous findings [19].

Our results suggest a model where Bod1 holds PP2A-B56 activity in check during prometaphase. This ensures that phosphorylation of Knl1, Ndc80 and other kinetochore substrates can occur, allowing turnover of attachments (figure 7*d*). Once amphitelic kinetochore–microtubule attachments are achieved, we observe rapid dephosphorylation of Bod1 at T95. We have previously shown that loss of Bod1 phosphorylation prevents its inhibition of PP2A-B56 [9]. Active PP2A-B56 can then dephosphorylate the SILK and RVSF motifs on KNL1 [15], resulting in recruitment of PP1 [12] and stabilization of kinetochore–microtubule attachments. Ndc80 dephosphorylation may be mediated by PP1 and/or PP2A directly, or indirectly through PP1 inhibition of Aurora B activation [13].

## 3. Discussion

Using a combination of proteomics, *in vitro* recombinant interaction studies and *in vivo* siRNA-mediated localization studies, we have demonstrated that the PP2A-B56 regulator Bod1 is recruited to kinetochores by Ndc80. Using quantitative immunofluorescence, we have also shown that Bod1 is required to prevent premature dephosphorylation of Ndc80 and Knl1 during early mitosis. Together with MCAK S92 [19] and CENP-U/PBIP1T78 [9], we add Ndc80 S55, Knl1 S24 (SILK), Knl1 S60 (RVSF) [5] and two of the multiple Knl1 phospho-MELT sites, T943 and T1155 [15,52], to the list of phosphoepitopes at kinetochores affected by Bod1 depletion. Bod1 therefore controls the phosphorylation of distinct groups of kinetochore proteins, all of which are implicated in the establishment of proper amphitelic kinetochore–microtubule attachments.

Bod1 is present at kinetochores throughout mitosis, from nuclear envelope breakdown until the end of anaphase [19]. Here, quantitative analysis has demonstrated that Bod1 localization to kinetochores peaks during metaphase. Phosphorylation of Bod1 at T95 is essential for its inhibitory function against PP2A-B56 [9]. In contrast to the total population of Bod1, the pool of phospho-T95 Bod1 peaks at kinetochores during prometaphase. This timing potentiates the inhibitory activity towards PP2A-B56 when phosphatase localization to kinetochores is greatest and PP2A activity needs to be properly regulated to allow correction of erroneous attachments [11].

We show here that Knl1 depletion reduces PP2A-B56 at kinetochores. This supports a growing body of evidence that a pool of PP2A-B56 is recruited to kinetochores in a Knl1/checkpoint protein-dependent manner [17,18]. However, our data suggest that there may also be other PP2A-B56 recruitment sites within the kinetochore, as PP2A-B56 levels can be partially rescued upon co-depletion of Bod1 with Knl1. This is supported by our previous observation that demonstrated there was no significant reduction in PP2A levels at kinetochores in metaphase cells [9]. The site of alternative PP2A-B56 recruitment remains to be identified.

Inter-complex interactions within the KMN network are an emerging theme in attachment regulation: the mitotic checkpoint kinase Mps1 is recruited to kinetochores through Ndc80 [14,53–57], but it phosphorylates MELT motifs on Knl1 [39–41]. Our data suggest that Bod1, also recruited through Ndc80, can regulate phosphatase activity toward Knl1 as well as Ndc80, providing another example of functionally important interactions between different components of the KMN network. The mechanisms that control these interactions remain to be determined.

## 4. Material and methods

### 4.1. Cell lines and cell culture

HeLa S3 cells were maintained in EMEM (Lonza), and supplemented with 10% fetal calf serum, 2 mM l-glutamine, 100 U ml^−1^ penicillin and 100 mg ml^−1^ streptomycin. A cell line stably expressing Bod1-GFP was generated using HeLa cells harbouring a single Flp recombination target site in their genome (a kind gift from Patrick Meraldi [58]) and maintained in the media described above with an additional 200 mg ml^−1^ hygromycin. Cells lines were maintained at 37°C with 5% CO_2_ in a humidified incubator. For transfections, cells were seeded in six-well dishes and transfected with 300 ng plasmid DNA per well using Effectene transfection reagent (Qiagen) or with 33 nM siRNA oligo duplexes or medium GC control siRNA (Invitrogen) using lipofectamine 2000 (Invitrogen). Cells were split onto coverslips the next day. Immunofluorescence staining of the cells and immunoblot analysis were performed 48 h after siRNA transfection. Small molecules used in this study were: Eg5 inhibitor *S*-trityl-l-cysteine (STLC) at 5 µM for 18 h. Cdk1 inhibitor RO-3306 at 10 µM for 10 min (acute Cdk1 inhibition) or overnight (G2/M arrest). Proteasome inhibitor MG132 at 10 µM for 30 min.

### 4.2. Generation of peptide antibodies

To raise the pT95 Bod1 phospho-antibody, sheep were immunized with the immunogenic phosphopeptide NH2-CRQKVDNFVS[pT]HLDKQ-COOH, comprising R86-Q100 of human Bod1. Serum containing the polyclonal antibody was collected in three batches. To raise the total Bod1 antibody, not directed against T95, sheep were immunized with a NH2-CRNGLRQSVVQS-COOH peptide, comprising R112-S122 of human Bod1. The third batch, obtained 91 days after the initial immunization and 7 days after the third antigen booster injection, was used for antibody purification. For phospho-antibody purification, the antibody was first purified using a non-phosphopeptide NH2-CRQKVDNFVSTHLDKQ-COOH column to deplete the serum of any pan-specific antibodies. The remainder of the serum was run over a column containing the phosphopeptide, yielding strictly phospho-specific antibodies. The total Bod1 antibody was purified using only the non-phosphopeptide column. To prepare the peptide-coated columns, 5 ml Affigel-10 (Bio-Rad) were activated by consecutive treatment with 5% ethylene diamine and 7 mg IAA-NHS ester. Five milligrams of the respective peptide in 0.1 M Na phosphate buffer pH7.8 was added to the fully activated resin overnight. Then, residual iodoacetate groups were blocked with 0.2% β-mercaptoethanol, and non-covalently bound peptide was removed by consecutive washes with 0.1 M NaHCO_3_, 1 M Na_2_CO_3_, water, 0.2 M glycine–HCl pH 2.0, 150 mM NaCl and TBS. The resin was stored in 0.1% NaN_3_ in TBS. For antibody purification, 4 ml serum was diluted 1 : 1 with TBS and passed through a 0.2 µm filter. The diluted serum was run over the peptide-coated column ten times. The column was then washed with TBS, 0.5 M NaCl, 20 mM Tris–HCl pH7.4, 0.2% Triton-X in TBS, and TBS. A low pH elution was performed with 0.15 M NaCl, 0.2 M glycine–HCl pH2.0 collecting 1 ml fractions with each tube containing 0.1 ml 2 M Tris–HCl pH8.5. After re-equilibrating the pH of the column by washing with TBS, a second, guanidinium hydrochloride elution was performed with 6 M guanidine hydrochloride in TBS. Samples of all fractions were spotted onto nitrocellulose membranes and protein content was visualized with Ponceau S (Sigma). All fractions that contained antibody proteins were pooled and dialysed into TBS overnight. Antibodies were stored in 0.1% sodium azide (NaN_3_) in TBS at 4°C.

### 4.3. Immunofluorescence and microscopy

Cells were seeded on coverslips (thickness 1.5) 24 h before fixation. Cells were pre-permeabilized with ice-cold cytoskeleton (CSK) buffer (100 mM NaCl, 300 mM sucrose, 3 mM MgCl_2_, 10 mM PIPES (pH6.8)) containing 0.1% Triton X-100 for 3 min at 4°C before fixation with 3.7% paraformaldehyde in PBS at room temperature. Samples were re-hydrated with TBS containing 0.1% Triton X-100 (TBS-T) before transferring coverslips into a moist chamber for blocking with 1% normal donkey serum in AbDil (0.25% v/v Tween-20, 2% w/v BSA, 0.1% w/v NaN_3_ in TBS). Primary antibodies were added, diluted in AbDil, for 1 h. Cells were carefully washed with TBS-T and secondary antibodies (1 : 500 in AbDil, Jackson ImmunoResearch) were added for 30 min in the dark. Cells were washed again and 4',6-diamidino-2-phenylindole (DAPI, Sigma) was added at 1 µg ml^−1^ in TBS for 10 min. Coverslips were washed with TBS and mounted onto microscope slides by inverting them into mounting medium (0.5% p-phenylenediamine (Free Base; Sigma) in 20 mM Tris, pH 8.8, 90% glycerol). Primary antibodies included: polyclonal sheep Bod1 antibodies (0.5 µg ml^−1^), mouse anti-B56α (1 : 100, BD Biosciences), mouse anti-Ndc80 (1 : 500, Abcam [9G3]), mouse anti-Nuf2 (1 : 300, Abcam), rabbit anti-CASC5 [Knl1] (1 : 1000, Abcam), mouse-anti-Plk1 (1 : 500, Upstate), rabbit anti-Ndc80 (phospho-Ser55) antibody (1 : 300, GeneTex), rabbit anti-Knl1 (phospho-Ser24) antibody (1 : 2000, a kind gift from Iain Cheeseman), rabbit anti-Knl1 (phospho-Ser60) antibody (1 : 2000, a kind gift from Iain Cheeseman), rabbit anti-Knl1 (phospho-Thr943/1155) antibody (1 : 1000, a kind gift from Adrian Saurin), rabbit anti- CENP-U/PBIP1 (phospho-Thr78) antibody (1 : 500, Abcam), rat anti-tubulin (1 : 500, AbD Serotec), human anti-centromere autoantisera [ACA] (1 : 1000, a kind gift from Sara Marshall, Ninewells Hospital, Dundee). Three-dimensional deconvolution image datasets were acquired on a DeltaVision imaging system (Applied Precision) equipped with an Olympus 1-UB836 microscope, CCD camera (CoolSNAP_HQ/ICX285), and 100×/1.4 NA Plan-Apochromat oil immersion objectives (Olympus). Z stacks were collected 0.2 µm apart to cover the full volume of DAPI-stained DNA within each mitotic cell and deconvolved using softWoRx (Applied Precision).

### 4.4. Image analysis

Image data were imported into OMERO and quantification of kinetochore intensities was performed using OMERO.mtools [59]. This image analysis is based on interrogating the data as a volumetric object and not maximum intensity projections. In brief, a cuboid region of interest was determined around the DAPI channel across *x*, *y* and *z*. Kinetochores within these regions were segmented in *x*, *y* and *z* based on anti-centromere antibody (ACA) staining using Otsu thresholding. To exclude noise, the minimum object size was set to 70 pixels. Because Bod1 kinetochore staining was mainly contained within the outer kinetochore, the perimeter of the ACA-based segmentation mask was expanded by 4 pixels (0.32 µm) to include the outer kinetochore in the analysis. The fluorescence signal within this mask was measured in each additional channel imaged. Background staining was quantified in a 2-pixel annulus with a 1-pixel gap to the perimeter of each segmented mask and the average background intensity was subtracted from each pixel within the mask. Fluorescence intensity at each kinetochore was then calculated as the summed fluorescence intensity within the ACA mask. All images were stored in OMERO, and figures were generated using OMERO.figure.

### 4.5. Affinity purification and immunoblotting

For affinity purification, HeLa S3 cells were arrested in mitosis by treatment with 5 µM STLC for 18 h. After gentle mitotic shake-off, cells were resuspended in lysis buffer (20 mM Tris acetate pH 7.5, 1 mM EGTA, 1 mM EDTA, 10 mM Na-β-glycerophosphate, 5 mM Na-pyrophosphate, 1 mM Na-orthovanadate, 50 mM NaF, 1 µM microcystin, 0.27 M sucrose, 10 µg ml^−1^ leupeptin, 10 µg ml^−1^ pepstatin, 10 µg ml^−1^ aprotinin), containing 0.01% and 0.05% Triton X-100 for stable and transient transfections, respectively, and disrupted with four rounds of freeze fracturing. After depleting insoluble proteins by centrifugation (4°C, 10 000 rpm, 5 min), affinity purification was performed using GFP Binder (Chromotek) for 90 min at 4°C and constant agitation. Purified samples were washed and resolved by SDS-PAGE. Immunoblotting was performed using standard procedures, and secondary antibody was detected using either Clarity Western ECL Substrate (Bio-Rad) and X-Ray films (Kodak) or the Odyssey Clx infrared detection system (LI-COR). Primary antibodies included mouse anti-B56α (1 : 500, Abcam), mouse anti-B56δ (1 : 500, Abcam), mouse anti-Ndc80 (1 : 1000, Abcam [9G3]), mouse anti-Nuf2 (1 : 1000, Abcam), rabbit anti-Spc24 (1 : 1000, Abcam [EPR11548(B)]), mouse anti-MBP (1 : 20 000, NEB), goat anti-GST (1 : 5000, Abcam), mouse anti-Vinculin (1 : 10 000, Abcam [SPM227]), mouse anti-GFP (1 : 1000, Roche), rabbit anti-Bod1 (1 : 500, Abcam), polyclonal sheep anti-Bod1 (2 µg ml^−1^). Secondary antibodies were sheep anti-mouse IgG, HRP-linked (1 : 10 000, GE Healthcare), goat anti-rabbit IgG, HRP-linked (1 : 5000, Cell Signalling), donkey anti-goat IgG, HRP-linked (1 : 20 000, Promega), donkey anti-sheep HRP (1 : 20 000, Sigma), IRDye 680LT donkey anti-mouse IgG (H + L) (1 : 20 000, LI-COR), IRDye 800CW donkey anti-goat IgG (H + L) (1 : 20 000, LI-COR). LI-COR images were quantified using ImageStudio software v. 2.0 (LI-COR), with signal intensity normalized to input protein levels.

### 4.6. Mass spectrometry

Eight 15 cm plates of stably Bod1-GFP or GFP transfected cells or two 15 cm plates of transiently Bod1-GFP or GFP transfected cells were arrested in mitosis, and affinity purification using GFP Binder (Chromotek) was performed as described above. Proteins were eluted with 2× SDS buffer and the full eluate was run on a 4–12% SDS-PAGE. Bands were visualized using Coomassie Brilliant Blue and lanes were cut into four gel pieces. Gel pieces were subsequently de-stained with ammonium bicarbonate and acetonitrile as an organic solvent and dried completely in a vacuum centrifuge. Cysteine disulfide bonds were reduced with 10 mM DTT and the resulting thiol groups were irreversibly alkylated to *S-*carboxyamidomethylcysteine with 55 mM iodoacetamide. Excess iodoacetamide was removed and gel pieces were dried in a vacuum centrifuge before enzymatic digest of the proteins. In-gel digest was performed with 20 ng µl^−1^ trypsin in 50 mM ammonium bicarbonate at 37°C o/n. Tryptic peptides were extracted from the gel by repeated addition of 0.1%TFA/acetonitrile extraction solution and sonication. Peptide samples were cleaned for mass spectrometry using a C18-Ziptip protocol. Mass spectrometry was performed on an LTQ Orbitrap Velos Pro instrument (Thermo Fisher Scientific). Mass spectrometry raw data were processed in the MaxQuant software package v. 1.3.0.5 utilizing the Uniprot Human database (09/08/2012) [60]. Parameters applied include: minimum peptide length = 7, protein FDR = 0.01, site FDR = 0.01. Peptides with variable modifications (N-terminal acetylation of the protein, oxMet, and pyroGlu) and fixed modifications (*S*-carboxyamidomethylcysteine) were accounted for in the analysis. Shotgun proteomics data analysis, including statistical analysis and GO term analysis, was performed using the Perseus software package v. 1.5.5.3 [61]. Statistical test performed was an unpaired Student's *t*-test with a threshold *p*-value of 0.05.

### 4.7. Protein expression and purification

Ndc80 Bonsai and recombinant Ndc80/Nuf2–GST or Spc24–GST/Spc25 were expressed and purified as described previously [37,38]. For production of Bod1-MBP and MBP, 5 ml LB medium containing the appropriate selection marker were inoculated with transformed BL21 *E. coli*. After 18 h at 37°C, starter cultures were transferred into 2 l conical flasks containing 500 ml LB medium with the selection antibiotic. Cultures were grown in shaking incubators at 37°C up to OD_600_ = 0.4. After adding 100 mM benzylalcohol for 30 min at 37°C, recombinant protein production was induced by addition of 0.1 mM isopropyl β-D-1-thiogalactopyranoside (IPTG). Protein expression was allowed for 18 h at 18°C. Bacteria were harvested by ultracentrifugation (5250*g*, 4°C, 30 min, slow deceleration) and lysed by resuspending them in PBS containing a protease inhibitor cocktail (Roche) and adding 1 mg ml^−1^ lysozyme. Cells were incubated at 4°C under constant agitation for 30 min after which Triton X-100 was added to a final concentration of 1%. The suspension was sonicated for 30 s on ice and left to incubate another 30 min at 4°C. The lysate was sonicated twice more and insoluble debris was pelleted by ultracentrifugation (26 000*g*, 4°C, 1 h). For protein purification, 1 ml amylose resin (NEB) was pre-equilibrated with binding buffer (50 mM Tris–HCl pH 7.5, 100 mM NaCl, 1 mM DTT) and the soluble fraction of the protein lysate was added after passing through a 0.2 µm filter. Binding was allowed for 2 h at 4°C under constant agitation. The recombinant protein bound to beads was washed with binding buffer. To elute the protein, 500 μl binding buffer containing 20 mM maltose were added and samples were incubated for 90 min at 4°C under agitation. The supernatant was transferred into a Slide-A-Lyzer dialysis cassette (Pierce) and dialysed into interaction buffer (20 mM Tris–HCl, 20 mM NaCl, 10% glycerol, 1 mM EGTA, 1 mM DTT) over night. The concentration of dialysed protein was determined using a Bradford colorimetric assay. If protein concentrations were below 0.2 mg ml^−1^, protein solutions were concentrated using Vivaspin columns (GE Healthcare) at 4750 rpm, 4°C. Proteins were aliquoted and stored at −80°C.

### 4.8. Pull down experiments

For *in vitro* binding studies, 150 pmol Ndc80Bonsai, coupled to glutathione beads, were pre-incubated with 0.01% insulin in interaction buffer (20 mM Tris–HCl, 20 mM NaCl, 10% glycerol, 1 mM EGTA, 1 mM DTT, Complete protease inhibitors (Roche)) for 20 min at 4°C. 1 nmol MBP or Bod1-MBP was added to the beads and binding was allowed to take place for 1 h at 4°C. After washing with interaction buffer, proteins were eluted with SDS loading buffer and all eluate was loaded for immunoblot analysis. Of note, 25 pmol MBP or Bod1-MBP were loaded as input controls. Band intensity was determined using the ImageStudio software package. Total amount of protein in the pull down was determined by using the input as a reference.

### 4.9. Statistical analysis

Statistical significance tests were performed using Sigma Plot v. 12.5 (Systat Software Inc.). For pairwise comparison, datasets were tested for normal distribution and then analysed by unpaired Student's *t*-test (for Gaussian distributions) or Mann–Whitney rank sum test (for non-Gaussian distributions). For group-wise comparison, datasets were compared by Kruskal–Wallis one-way analysis of variance (ANOVA) on ranks, followed by pairwise multiple comparison procedures (Dunn's Method).

### 4.10. siRNAs

Knl1 was depleted using 5′-GCAUGUAUCUCUUAAGGAA-3′ [15]. siRNA targeting Bod1 was 5′-GCCACAAAUAGAACGAGCAAUUCAU-3′ [19]. Ndc80 was depleted using an siRNA with the sequence 5′-AAGTTCAAAAGCTGGATGATCTT-3′ [62]. BubR1 was depleted using 5′-AGAUCCUGGCUAACUGUUC-3′ [15]. All isoforms of the B56 PP2A regulatory subunit were depleted using a pool of 5′-GCUCAAAGAUGCCACUUCA-3′ (B56α/PPP2R5A), 5′-CGCAUGAUCUCAGUGAAUA-3′ (B56β(PPP2R5B)), 5′-GGAUUUGCCUUACCACUAA-3′ (B56γ/PPP2R5C), 5′-UCCAUGGACUGAUCUAUAA-3′ (B56δ/PPP2R5D), 5′-UUAAUGAACUGGUGGACUA-3′ (B56*ɛ*/PPP2R5E), described in [11]. Stealth RNAi siRNA Negative Control, Med GC (Invitrogen) was used for control transfections.

## Supplementary Material

Supplementary figures S1-S5

## Supplementary Material

Table S1

## Supplementary Material

Table S2

## Data accessibility

The mass spectrometry proteomics data have been deposited to the ProteomeXchange Consortium [63] via the PRIDE partner repository [64] with the dataset identifier PXD006322. Original microscopy data can be accessed at http://dx.doi.org/10.17867/10000109.
