# Supplementary material for: The Ndc80 complex targets Bod1 to human mitotic kinetochores
Source: Open Biol. 2017 Nov 15;7(11):170099. doi: 10.1098/rsob.170099 (PMC5717335; doi:10.1098/rsob.170099)
Supplement: Supplementary figures S1-S5 [file rsob170099supp1.pdf]

Schleicher et al., Figure S1

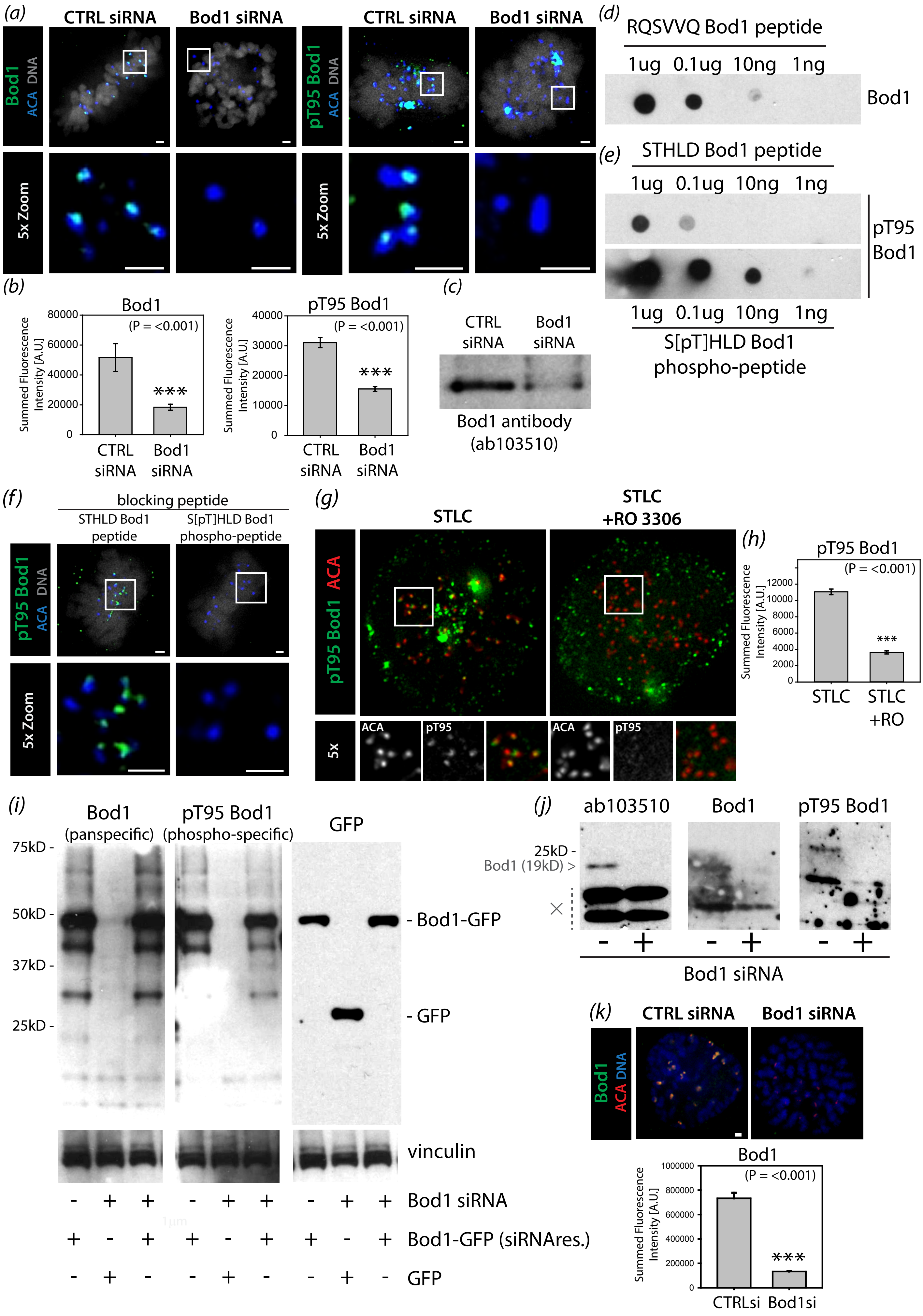

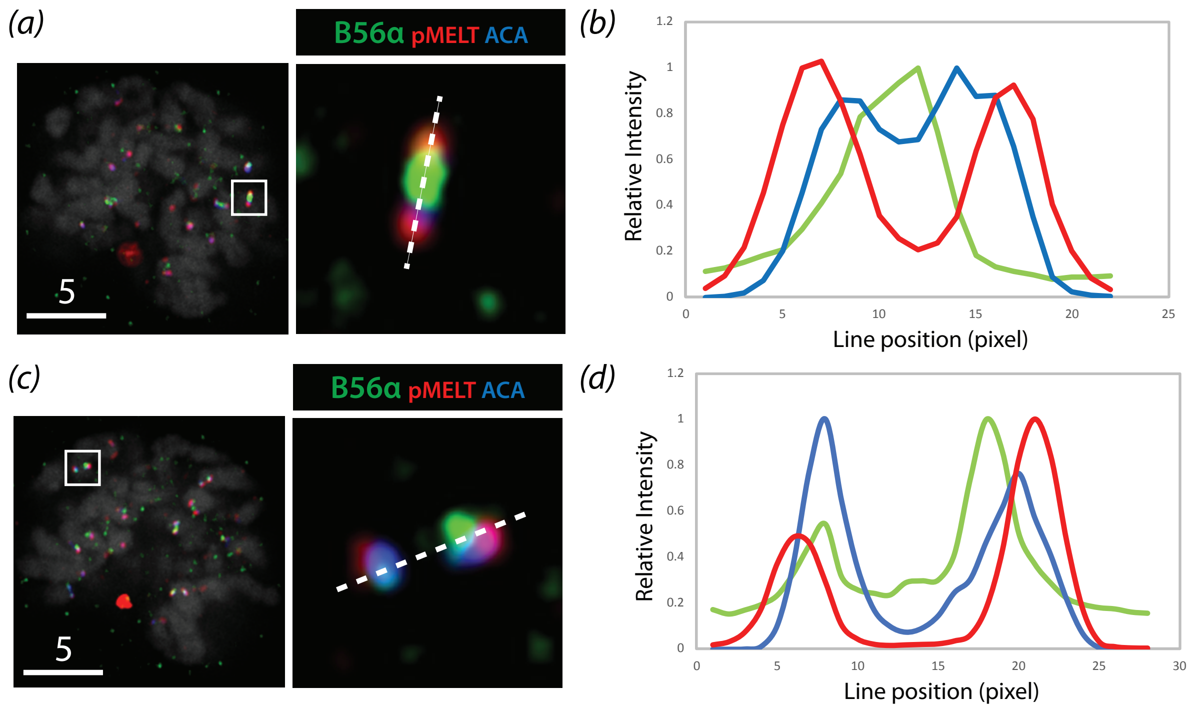

(a)
B56α pMELT ACA
5
(b)
Relative Intensity
Line position (pixel)
(c)
B56α pMELT ACA
5
(d)
Relative Intensity
Line position (pixel)

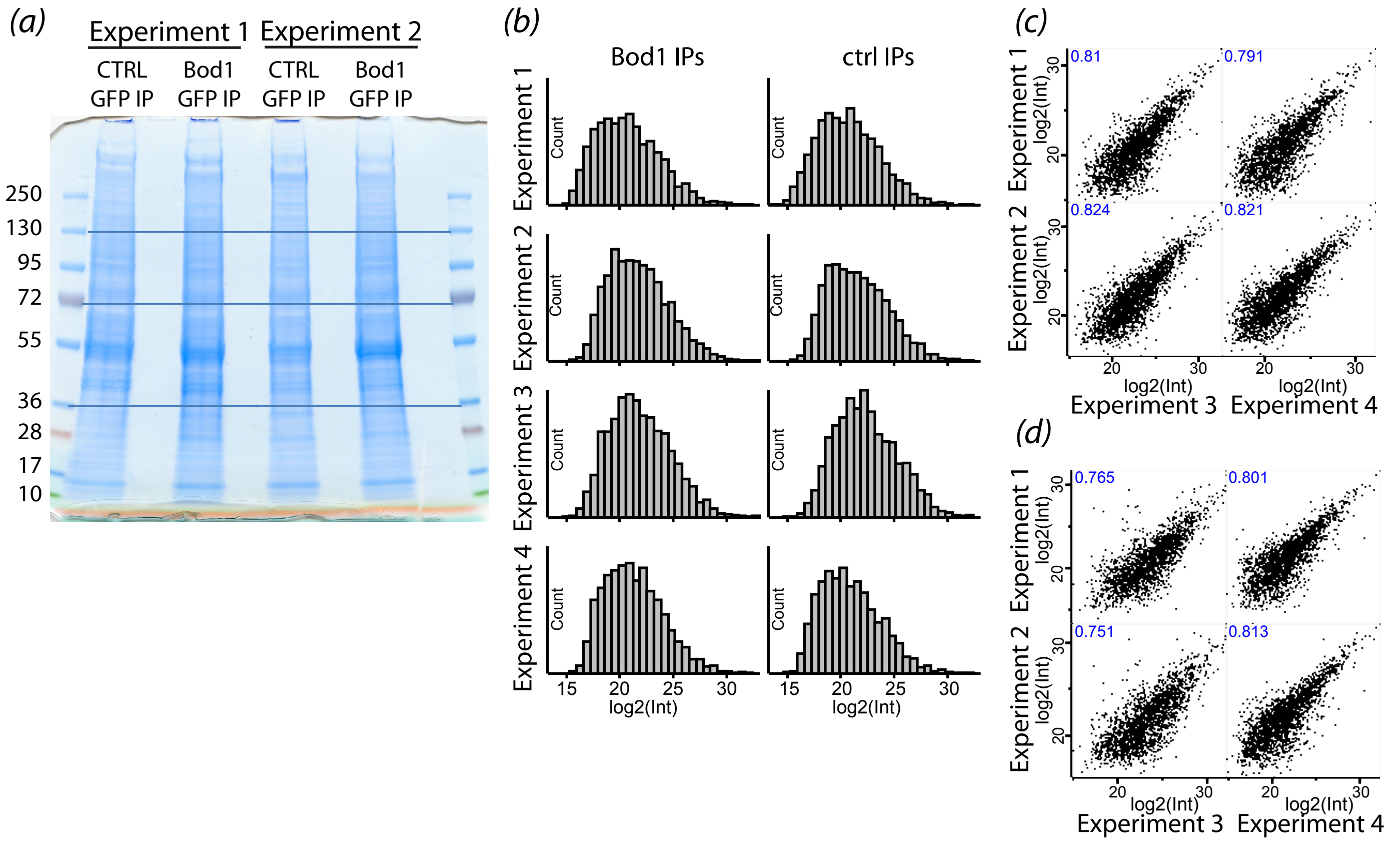
(a)
Experiment 1
Experiment 2
CTRL GFP IP
Bod1 GFP IP
CTRL GFP IP
Bod1 GFP IP
250
130
95
72
55
36
28
17
10
(b)
Bod1 IPs
ctrl IPs
Experiment 1
Experiment 2
Experiment 3
Experiment 4
Count
15 20 25 30
log2(Int)
(c)
0.81
0.791
0.824
0.821
Experiment 1
Experiment 2
log2(Int)
Experiment 3
Experiment 4
(d)
0.765
0.801
0.751
0.813
Experiment 1
Experiment 2
log2(Int)
Experiment 3
Experiment 4

(a)

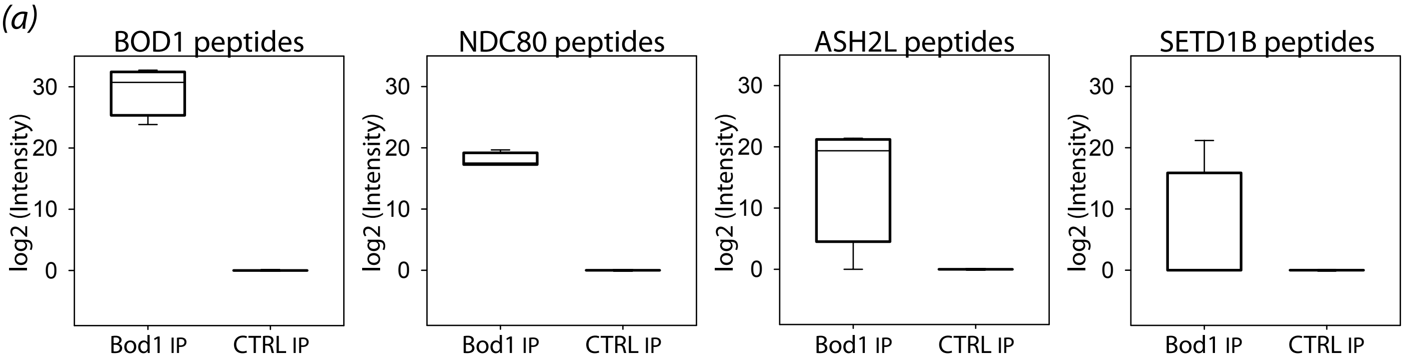
BOD1 peptides
NDC80 peptides
ASH2L peptides
SETD1B peptides
log2 (Intensity)
30
20
10
0
Bod1 IP
CTRL IP

(b)

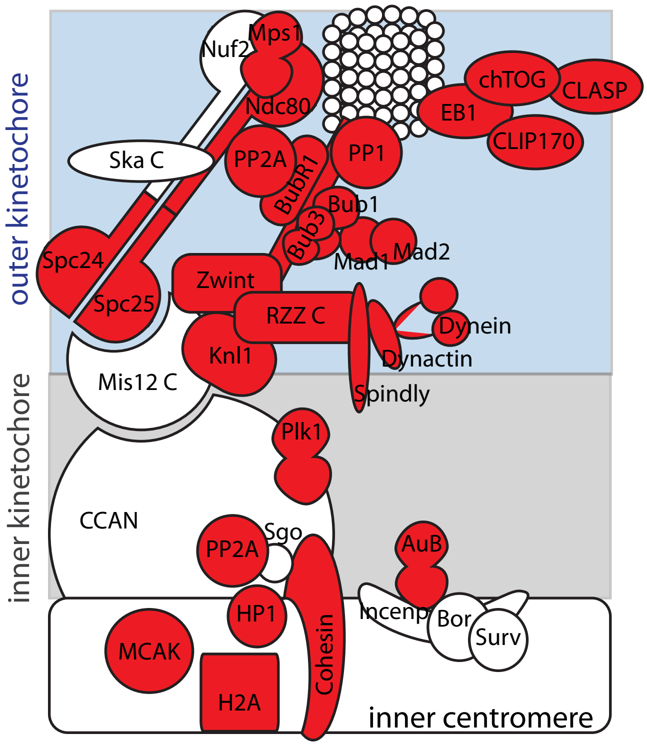
outer kinetochore
inner kinetochore
inner centromere
Nuf2
Mps1
Ndc80
chTOG
CLASP
EB1
CLIP170
Ska C
PP2A
BubR1
PP1
Bub1
Bub3
Mad1
Mad2
Spc24
Spc25
Zwint
RZZ C
Dynein
Dynactin
Knl1
Mis12 C
Spindly
Plk1
CCAN
PP2A
Sgo
AuB
HP1
Incenp
Bor
Surv
MCAK
H2A
Cohesin

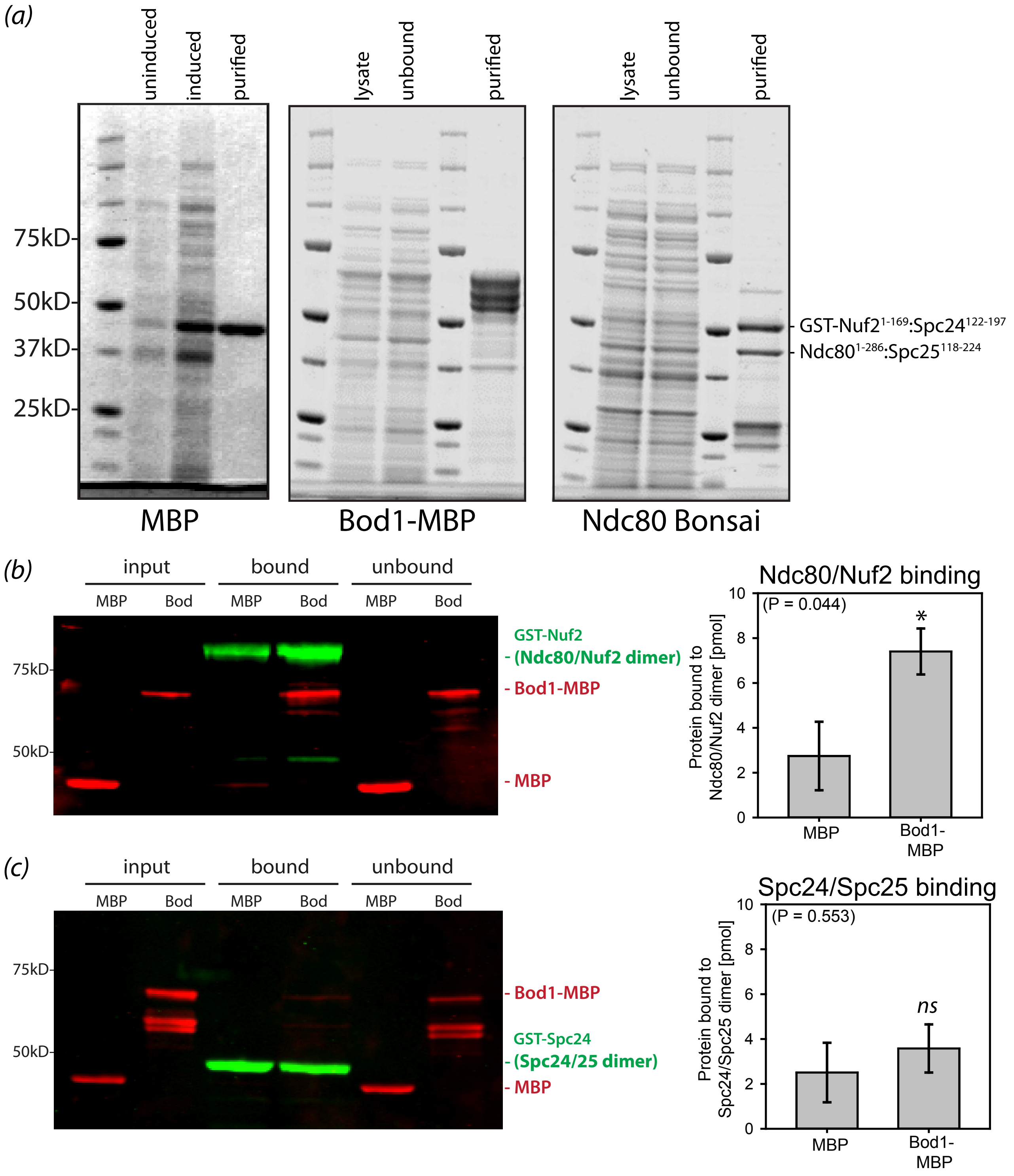
(a)
uninduced
induced
purified
lysate
unbound
purified
lysate
unbound
purified
75kD
50kD
37kD
25kD
GST-Nuf2 1-169:Spc24 122-197
Ndc80 1-286:Spc25 118-224
MBP
Bod1-MBP
Ndc80 Bonsai
(b)
input
bound
unbound
MBP
Bod
MBP
Bod
MBP
Bod
75kD
50kD
GST-Nuf2
(Ndc80/Nuf2 dimer)
Bod1-MBP
MBP
Ndc80/Nuf2 binding
(P = 0.044)
*
Protein bound to Ndc80/Nuf2 dimer [pmol]
0
2
4
6
8
10
MBP
Bod1-MBP
(c)
input
bound
unbound
MBP
Bod
MBP
Bod
MBP
Bod
75kD
50kD
Bod1-MBP
GST-Spc24
(Spc24/25 dimer)
MBP
Spc24/Spc25 binding
(P = 0.553)
ns
Protein bound to Spc24/Spc25 dimer [pmol]
0
2
4
6
8
10
MBP
Bod1-MBP

Supplementary Figure Legends

Figure S1. Extensive characterisation of Bod1-specific peptide antibodies in immunofluorescence and immunoblots. (*a*) HeLa cells were treated with control or Bod1 siRNA for 48h. They were fixed in paraformaldehyde and stained with either the total Bod1-specific peptide antibody (left panels) or the pT95 Bod1 phospho-specific peptide antibody (right panels) as well as ACA (blue) and DAPI (grey). Bottom panels are magnifications of the indicated section of the same cell (white boxes). (*b*) Kinetochore intensity of antibody staining was determined. (*c*) Efficiency of Bod1 depletion in the same experiment was determined using immunoblotting with the commercial Bod1 antibody ab103510. (*d*) Dilution series of the total Bod1 antigenic peptide NH2-CRNGLRQSVVQS-COOH (R112–S122 of human Bod1) applied to a nitrocellulose membrane was used for a dot blot test of total Bod1 antibody affinity. (*e*) Dilution series of the pT95 Bod1 antigenic phosphopeptide NH2-CRQKVDNFVS[pT]HLDKQ-COOH (R86–Q100 of human Bod1) and corresponding non-phosphopeptide applied to a nitrocellulose membrane were used for a dot blot test of pT95 phospho-specific Bod antibody affinity. (*f*) The pT95 phospho-specific Bod1 peptide antibody was pre-treated with either the antigenic phosphopeptide or the corresponding non-phosphopeptide and then used in immunostaining of paraformaldehyde-fixed HeLa cells. Bottom panels are magnifications of the indicated section of the same cell (white boxes). (*g*) HeLa cells were treated with 5µM of the Eg5 inhibitor STLC over night to arrest them in prometaphase and maximise T95 phosphorylation. To test Cdk1-dependency of pT95 Bod1 phosphorylation, the Cdk1 inhibitor RO-3306 was applied at 10µM for 10min. Bottom panels are magnifications of the indicated section of the same cell (white boxes). Maximum Intensity projection of 7 consecutive z-slices taken 0.2µm apart is shown. (*h*) Kinetochore intensity of pT95 Bod1 staining was determined. (*i*) Bod1 panspecific and pT95 phospho-specific peptide

antibodies purified from sheep serum were used for immunoblots. Asynchronous whole cell lysates of HeLa cells overexpressing siRNA resistant Bod1-GFP or GFP were prepared as input. Vinculin was used as loading control. (*j*) Immunoblots of endogenous Bod1 from whole cell asynchronous HeLa cell lysates. Membranes were cropped at 30kD before incubation with the antibody to maximise affinity to the Bod1-specific band. An Abcam commercial antibody (ab103510) as well as total and pT95 Bod1 peptides antibodies were used. Cross indicates non-specific bands. (*k*) HeLa cells were treated with control or Bod1 siRNA for 48h. They were fixed in paraformaldehyde and stained with the total Bod1-specific peptide antibody. Prophase cells were imaged and Bod1 kinetochore intensity was determined. Unless otherwise indicated, single z-sections are shown. Scale bars are 1 µm. Pairwise comparisons were evaluated by unpaired Student’s t-test. Two-tailed p-values are shown. n = 10 cells per condition. Error bars represent standard error.

Figure S2. Localisation of PP2A-B56α to inner and outer kinetochore compartments. HeLa cells were fixed in paraformaldehyde and stained with a PP2A-B56α isoform-specific antibody (green), a marker for the centromeric region (ACA, blue), and a marker for the outer kinetochore (pMELT, red). (*a*) Prometaphase cell with maximal centromeric PP2A-B56α targeting was imaged. The last panel is a magnification of the same cell (section indicated by white box). Single z-sections are shown. Scale bars are 5 µm. (*b*) Normalised line intensity profile through dashed white line in (*a*). (*c*) Prometaphase cell with maximal PP2A-B56α kinetochore targeting was imaged. Last panel is a magnification of the same cell (section indicated by white box). Single z-sections are shown. Scale bars are 5 µm. (d) Normalised line intensity profile through dashed white line in (*c*).

Figure S3. Mass spectrometry experiment to find Bod1 interaction partners at the kinetochore. (*a*) Coomassie gel of the first two biological replicates of Bod1-GFP and GFP control affinity purifications analysed by mass spectrometry. Blue horizontal lines indicate where the gel was cut to fractionate each lane into four gel pieces. (*b*) Histograms for the intensity distribution of each experiment. (*c*) Intensity correlation between biological replicates of Bod1-GFP affinity purifications. Pearson correlation coefficient is shown in blue. (*d*) Intensity correlation between biological replicates of control GFP affinity purifications. Pearson correlation coefficient is shown in blue.

Figure S4. Supplementary analysis of the mitotic Bod1 interactome. (*a*) Box-and-whisker-plots of selected peptide intensities quantified by mass spectrometry in Bod1-GFP and control samples. n = 4 biological replicates. Statistical test used was an unpaired Student's t-test. Whiskers extend from minimum to maximum. (*b*) Graphic representation of well-characterised kinetochore and centromeric proteins. Proteins quantified in at least one of the four Bod1-GFP affinity purifications are coloured in red.

Figure S5. *In vitro* pull down experiment to confirm interaction between Bod1 and Ndc80. (*a*) Coomassie gels showing induction and purification of the indicated proteins from BL21 cells. (*b, c*) 150pmol recombinant Ndc80/Nuf2-GST dimer (*b*), or Spc24-GST/Spc25 dimer (*c*) [1], subcloned in a modified pGEX-6P vector (pGEX-6P-2RBS) to support dicistronic expression, were expressed in BL21 cells and immobilised on Sepharose beads. They were then incubated with 1nmol Bod1-MBP or MBP. Binding was allowed for 1h. Proteins were resolved by SDS-PAGE and immunoblotted using simultaneous detection of the MBP (red) epitope tag on Bod1 and the GST (green) epitope tag on Nuf2 or Spc24. Amount of bound protein was quantified relative to the input. Asterisk represents significant difference in unpaired t-test. Two-tailed p-value is indicated. n = 4 separate experiments. Error bars represent standard error.

Table S1. List of all proteins significantly enriched in Bod1-GFP affinity purifications. This table includes all proteins detected by mass spectrometry that were significantly enriched in Bod1 samples as identified by an unpaired Student's t-test with a threshold p-value of 0.05. Significance is indicated with a "+" in the column "Student's T-test Significant". Degree of enrichment is displayed in column "log2 (fold Change Bod1/ctrl)", where high values correspond to a high degree of enrichment in Bod1-GFP samples.

Table S2. List of all kinetochore and centromeric proteins detected as interactors of Bod1-GFP in mitotic HeLa cells. Table includes all proteins that were quantified in at least one of the four in Bod1-GFP affinity purifications in the experiment described in figure 3 and had a GO annotation for either "kinetochore" or "centromeric region". Significant interactors, as identified in an unpaired Student's t-test with a threshold p-value of 0.05, are indicated with a "+" in the column "Student's T-test Significant".

Supplementary references:

1. Ciferri, C. et al. 2005 Architecture of the human Ndc80-Hec1 complex, a critical constituent of the outer kinetochore. *J. Biol. Chem.* **280**, 29088–29095. (doi:10.1074/jbc.M504070200)
